# Molecular and functional characterization of voltage-gated sodium channels in human sperm

**DOI:** 10.1186/1477-7827-7-71

**Published:** 2009-07-16

**Authors:** Francisco M Pinto, Cristina G Ravina, Manuel Fernández-Sánchez, Manuel Gallardo-Castro, Antonio Cejudo-Román, Luz Candenas

**Affiliations:** 1 Instituto de Investigaciones Químicas, CSIC, Avda. Americo Vespucio 49, 41092 Sevilla, Spain; 2 IVI-Sevilla, Avenida Republica Argentina 58, 41011 Sevilla, Spain

## Abstract

**Background:**

We have investigated the expression of voltage-gated sodium channels in human spermatozoa and characterized their role in sperm motility.

**Methods:**

Freshly ejaculated semen was collected from thirty normozoospermic human donors, with each donor supplying 2 different samples. Reverse transcription-polymerase chain reaction (RT-PCR) and immunofluorescence techniques were used to detect the mRNAs and proteins of interest. Sperm motility was measured by a computer-assisted sperm analysis system (CASA). Cytosolic free calcium was determined by fluorimetry in cells loaded with the fluorescent calcium indicator Fura-2.

**Results:**

The mRNAs that encode the different Nav alpha subunits (Nav1.1-1.9) were all expressed in capacitated human spermatozoa. The mRNAs of the auxiliary subunits beta1, beta3 and beta4 were also present. Immunofluorescence studies showed that, with the exception of Nav1.1 and Nav1.3, the Nav channel proteins were present in sperm cells and show specific and different sites of localization. Veratridine, a voltage-gated sodium channel activator, caused time- and concentration-dependent increases in progressive sperm motility. In sperm suspensions loaded with Fura-2, veratridine did not modify intracellular free calcium levels.

**Conclusion:**

This research shows the presence of voltage-gated sodium channels in human sperm and supports a role for these channels in the regulation of mature sperm function.

## Background

Voltage-gated sodium channels (VGSCs) play an essential role in the generation of the rapid depolarization during the initial phase of the action potential in excitable cells [1,2]. These complex membrane proteins are composed of an α and one or more auxiliary β subunits [2,3]. The α subunits are large proteins with a high degree of amino acid sequence identity; they contain an ion-conducting aqueous pore and can function without the β subunit as a Na^+ ^channel [2-4]. Nine different voltage-dependent Na^+ ^channel α subunits have been cloned in mammals, each of which is encoded by a different gene [5]. They can be further characterized by their sensitivity to the highly selective blocker tetrodotoxin (TTX). The TTX-sensitive α subunits are inhibited by TTX in the nanomolar range and include SCN1A (also known as Na_v_1.1), SCN2A (also known as Na_v_1.2), SCN3A (also known as Na_v_1.3), SCN4A (also known as Na_v_1.4), SCN8A (also known as Na_v_1.6), and SCN9A (also known as Na_v_1.7). The TTX- resistant α subunits are inhibited by TTX in the micromolar range and include SCN5A (also known as Na_v_1.5), SCN10A (also known as Na_v_1.8), and SCN11A (also known as Na_v_1.9) [2,5]. A tenth, related, nonvoltage-dependent atypical α isoform, SCN7A (also known as Na_x_), has also been cloned and expressed [6,7]. Four different β subunits, SCN1B, SCN2B, SCN3B, and SCN4B (also named β_1–4_) are currently known [8-10]. The roles of the β subunits are less well established, although they appear to modulate the cellular localization, functional expression, kinetics, and voltage-dependence of channel gating [8,10].

In mammalian spermatozoa the acquisition of fertilization competence, known as capacitation, occurs during the transit through the female reproductive tract and is accompanied by important changes in sperm motility, intracellular pH (pH_i_) and plasma membrane potential (E_m_) and organization [11-16]. In addition to the pivotal role played by Ca^2+ ^[17], Na^+ ^and K^+ ^fluxes through plasma membrane may contribute specially to these processes, necessary for the morphological and functional changes of sperm that ultimately lead to interaction with the oocyte [11,14,18,19]. Molecular and functional studies of K^+ ^channels have revealed that voltage-gated K_v _channels, Ca^2+^-activated K^+ ^channels and inwardly rectifying K_ATP _channels are present and have a potential functional role in sperm [14,20]. Regarding Na^+ ^channels, Hernández-González et al. [19] reported the involvement of an amiloride-sensitive Na^+ ^channel that may contribute to the regulation of resting sperm E_m_. The characteristics of these channels match with the family of epithelial Na^+ ^channels (ENaC). Conversely, no studies have been made to characterize the presence of VGSCs in mature spermatozoa.

The major aim of our study was to characterize the presence and function of voltage-dependent Na^+ ^channels in capacitated human sperm. For this purpose, we analyzed the expression and localization of VGSC and realized experiments to investigate the effects of the selective VGSC activator veratridine on sperm motility.

## Methods

### Semen samples and sperm preparation

This study was approved by the Ethics Committees of CSIC and Instituto Valenciano de Infertilidad, Sevilla, and all donors gave written informed consent.

Freshly ejaculated semen was collected from 30 donors (18–35 years old) with normal sperm parameters and proven fertility. Samples (2 from each donor) were obtained by masturbation after 3–4 days sexual abstinence and processed immediately upon liquefaction. Quantitative, manual semen analyses were performed on undiluted semen (5 μl) with a Makler Counting Chamber (Sefi Medical Instruments, Haifa, Israel). Samples were examined for concentration and motility according to the World Health Organization (WHO, 1999) guidelines. A minimum of 200 cells were counted per 5 μl drop, and at least two drops were studied per sample.

Liquefied semen samples were washed with modified human tubal fluid (mHTF) supplemented with 2% bovine serum albumin (BSA) at 37°C and processed for capacitation as previously described [21]. Briefly, sperm suspensions were centrifuged at 400 *g *for 20 min through a discontinuous Percoll density gradient (Spermgrad-125, Vitrolife, Kungsbacka, Sweden). The samples were then centrifuged (400 *g *for 15 min), and the pellets collected and washed (400 *g *for 5 min) in 2 ml of mHTF. Samples were allowed to swim-up for 1 h at 37°C and the supernatant carefully aspirated. Semen motility and concentration were re-examined and the sperm concentration adjusted to 50 × 10^6 ^cell/ml for subsequent experiments.

### RNA extraction and RT-PCR

Total RNA from human sperm was extracted using TriReagent (Sigma). The complementary DNA (cDNA) was synthesized using the Quantitect Reverse Transcription kit (Qiagen, Venlo, The Netherlands). Human testis cDNA was obtained from Clontech (Palo Alto, CA, USA). The specific oligonucleotide primers designed to amplify the different voltage-dependent Na^+ ^channels α and β subunits have been previously used to investigate the expression of VGSC in 20 different human tissues [9] and span at least one exon in each target gene. Primers were also designed to amplify β-actin (*ACTB*), which was chosen as a housekeeping gene to control RT-PCR reactions among samples [21,22]. Amplification was carried out in 25 μl of PCR buffer containing 3 μl of cDNA reaction mixture, 2.5 mM MgCl_2_, 0.2 μM primers, 200 μM dNTPs and 1.5 U of heat-activated thermostable DNA polymerase (Immolase, Bioline, London, UK). PCR was performed for 35 cycles with cycling parameters of 15 s at 94°C, 20 s at 60°C and 20 s at 72°C. The PCR products were separated by agarose gel electrophoresis and the amplicon sizes were verified by comparison with a DNA mass ladder. The identity of each product was established by DNA sequence analysis. Each assay was performed in triplicate and three negative controls were run for each assay: no template, no reverse transcriptase and no RNA in the reverse transcriptase reaction.

### Immunofluorescence studies

Sperm cells were washed, resuspended in phosphate-buffered saline (PBS) and smeared onto poly-L-lysine-coated slides. Spermatozoa were then fixed by incubation in cold methanol (-20°C) for 20 min. Slides were washed three times for 10 min with PBS and incubated with 2% BSA in PBS for 30 min to block non-specific sites. Test slides were incubated with a primary polyclonal antibody designed to recognize Na_v_1.1 (sc-16031, goat), Na_v_1.2 (sc-28753, rabbit), Na_v_1.3 (sc-22202, goat), Na_v_1.4 (sc-28751, rabbit) and Na_v_1.5 (sc-22758, rabbit), from Santa Cruz Biotechnology (Santa Cruz, CA); Na_v_1.6 (asc-009, rabbit) from Alomone Labs (Jerusalem, Israel); Na_v_1.7 (ab-65167, rabbit), Na_v_1.8 (ab-66743, rabbit), Na_v_1.9 (ab-65160, rabbit) and Na_x _(ab-66499, rabbit), from Abcam (Cambridge, UK). All these primary antibodies were diluted 1:100 in PBS containing 2% BSA and incubated overnight at 4°C. The specificity of antibodies was assessed by the supplier or by pre-absorption with the corresponding immunogenic peptide when available. Negative control slides were not exposed to the primary antibody and were incubated with a) rabbit or goat IgG fraction or b) PBS and then processed in the same conditions as the test slides. Samples were washed three times in PBS, and incubated for 60 min with appropriate FITC-conjugated secondary antibodies (Santa Cruz). Slides were further washed in PBS, mounted using Vectashield (Vector Laboratories, Burlingame, CA) and examined with a Olympus BX-51 fluorescence microscopy (Tokyo, Japan) using a 100× immersion objective.

### Human sperm motility studies

Motility analysis was conducted by computer-assisted sperm analysis (CASA) (Sperm Class Analyzer, S.C.A., Microptic, Barcelona, Spain). Setting parameters and the definition of measured sperm motion parameters for CASA were established by the manufacturer: number of frames to analyze: 25; number of frames/s: 25; straightness (STR) threshold: 80%; cell size range (low): 2; cell size range (high): 60; volume ≥ 3.0 ml; sperm concentration/ml ≥ 20 × 10^6 ^cell/ml; forward motility ≥60%. To measure both sperm concentration and motility, aliquots of semen samples (7.5 μl) were placed into a pre-warmed (37°C) Makler counting chamber (Sefi Medical Instruments, Haifa, Israel). A minimum of 100 sperm from at least two different drops of each sample was analyzed from each specimen. The motility pattern of sperm samples was established following WHO guidelines and defined as: "A" grade sperm (rapidly progressive with velocity 25 μm/s), "B" grade (slow/sluggish progressive with velocity 5 μm/s but < 25 μm/s), "C" grade (non-progressive motility with velocity < 5 μm/s) and "D" grade (immobile) [22-24]. Progressive motility (*A *+ *B*), non-progressive motility (*C*) and immotility (D) were measured as percentage of the total (*A+B+C+D*) that was considered as 100%. All samples used in this study had values of immotile, grade *D *spermatozoa lower than 20% of the total.

To investigate the effects of veratridine, individual sperm samples were divided in several aliquots and each aliquot was treated with a single concentration of veratridine (10^-8 ^M, 10^-7 ^M, 3 × 10^-7 ^M, 10^-6 ^M, 3 × 10^-6 ^M, 10^-5 ^M or 3 × 10^-5 ^M) or the corresponding solvent (time-matched paired controls). Sperm motility was measured 5 min before agent addition (initial value) and after a contact time of 2, 15, 30 and 60 min. Values of sperm progressive motility, non-progressive motility and immotility were expressed as the positive or negative percentage increment in motility produced by the drug relative to the value observed at the same time in solvent-treated time-matched paired controls (Δ sperm motility).

### Measurements of [Ca^2+^]_i_

After capacitation and swim-up, spermatozoa were incubated with the acetoxymethyl ester form of Fura-2 (Fura-2/AM, 8 μM, Molecular Probes, Invitrogen, Eugene, OR, USA) for 60 min at room temperature. After loading, the cells were washed, resuspended in HEPES solution and used within the next 2–4 hours, following previously published procedures [25]. Sperm aliquots (1 ml, 50 × 10^6 ^cell/ml) were placed in the quartz cuvette of a spectrofluorometer (SLM Aminco-Bowman, Series 2, Microbeam, Barcelona, Spain) and magnetically stirred at 37°C. The sperm suspension was alternatively illuminated with two excitations wavelengths (340 nm and 380 nm) and the emitted fluorescence was measured at 510 nm. Changes in [Ca^2+^]_i _were monitored using the Fura-2 (F340/F380) fluorescence ratio as previously described [26,27].

### Drugs and solutions

The modified human tubal fluid was from Irvine Scientific (Santa Ana, CA, USA). The composition of the HEPES solution was (in mM): NaCl 140; KCl 4.7; CaCl_2 _2.0; MgCl_2 _0.3; glucose 10 and HEPES 10 (pH 7.4). Veratridine was from Sigma. Veratridine was dissolved in DMSO at a concentration of 10^-2 ^M, aliquoted and stored at -20°C until use. Further dilutions were made in mHTF or HEPES solution on the day of use.

### Statistical analysis

Values (means ± SEM) were obtained by pooling individual data. Unless otherwise indicated, *n *represents the number of experiments in sperm samples from *n *different donors. Multiple means were compared by one-way analysis of variance (ANOVA) followed by Newman-Keuls multiple comparison test. These procedures were undertaken using GRAPHPAD PRISM (version 5.0) program. A value of *P *< 0.05 was considered significant.

## Results

### mRNA expression of voltage-gated Na^+ ^channels in human sperm

**Figure 1:**
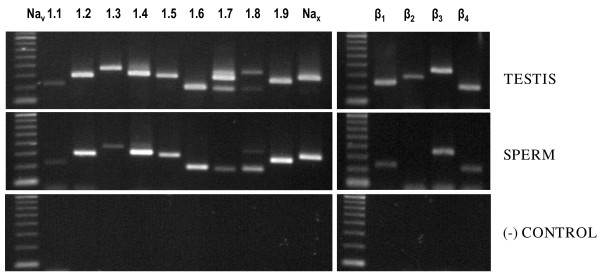
**Voltage-gated Na^+ ^channel gene expression in human sperm**. Agarose gel showing expression of messenger RNA for voltage-gated Na^+ ^channel α and β subunits and Na_x _in human testis and sperm. The specific bands were not detected in the negative controls and the one with no added RNA in the reverse transcriptase reaction is shown. M, molecular weight standards.

The PCR products expected for the Na_v _α subunits *SCN1A *(Na_v_1.1, 225 bp), *SCN2A *(Na_v_1.2, 297 bp), *SCN3A *(Na_v_1.3, 367 bp), *SCN4A *(Na_v_1.4, 317 bp), *SCN5A *(Na_v_1.5, 294 bp), *SCN8A *(Na_v_1.6, 207 bp), *SCN9A *(Na_v_1.7, 289 bp), *SCN10A *(Na_v_1.8, 347 bp), *SCN11A *(Na_v_1.9, 272 bp) and the related isoform *SCN7A *(Na_x_, 327 bp) were all expressed in human sperm cDNA (Fig 1). Splice variants of Na_v_1.7 and Na_v_1.8, that resulted from the deletion of exon 5 and 8, respectively, were also observed (Fig. 1). In the case of Na_v_1.7, both the wild type and the splice variant were detected in some sperm samples while only the splice variant was present in other sperm cDNAs (see Fig. 1) The mRNAs of the Na_v _β subunits *SCN1B *(β_1_, 236 bp), *SCN3B *(β_3_, 346 bp), and *SCN4B *(β_4_, 217 bp), and of the reference gene *ACTB *(362 bp) were also detected (Fig. 1, not shown for *ACTB*). All PCR products expressed in sperm cDNA were present in human testis cDNA (Fig. 1).

No PCR products were detected in the three negative controls showing the absence of genomic DNA contamination and that reagents were free of target sequence contamination. Fig. 1 shows the negative control with no RNA in the reverse transcriptase reaction.

### Immunodetection of voltage-gated Na^+ ^channel proteins in human sperm

**Figure 2:**
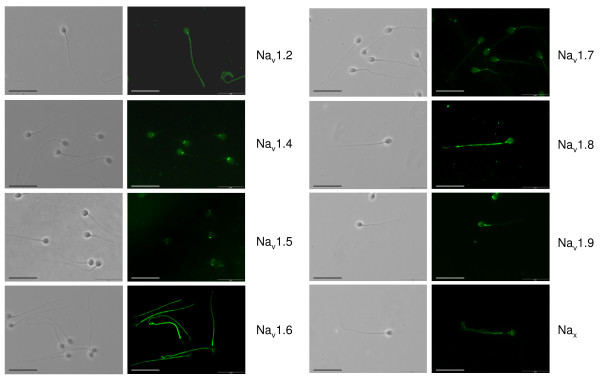
**Immunofluorescent localization of voltage-gated Na^+ ^channels in human sperm**. Immunofluorescence and corresponding phase-contrast images of sperm cells stained with primary antibodies against Na_v_1.1–1.9 and Na_x _showing specific localizations for each Na^+ ^channel. Experiments were performed at least six times for each channel with similar results. Scale bar: 20 μM.

Immunofluorescence analysis demonstrated that most Na_v _α subunits were present in the plasma membrane of human spermatozoa, and showed specific and different sites of localization (Fig. 2). SCN2A (Na_v_1.2) immunostaining was located in the flagellum and over the post-equatorial region of the head. SCN4A (Na_v_1.4) was present over the head and the flagellum midpiece, and the most intense immunofluorescence was detected in the post-acrosomal region around the connecting piece. SCN5A (Na_v_1.5) staining was strictly localized in the post-equatorial region of the head. SCN8A (Na_v_1.6) was present in the flagellum principal piece with a faint fluorescence staining being observed around the neck. SCN9A (Na_v_1.7) was mainly found around the connecting piece with a less intense immunostaining of the sperm head and flagellum. A strong positive signal for SCN10A (Na_v_1.8) was observed along the tail and around the connecting piece, with a less intense signal detected in the sperm head, mostly in the post-equatorial region. SCN11A (Na_v_1.9) was present over the acrosomal region of the head and in the flagellum midpiece. The atypical isoform SCN7A (Na_x_) was present in the flagellum and in the equatorial segment of the head (Fig. 2). The antibodies for SCN1A (Na_v_1.1) and SCN3A (Na_v_1.3) did not give any positive signal (not shown).

**Figure 3:**
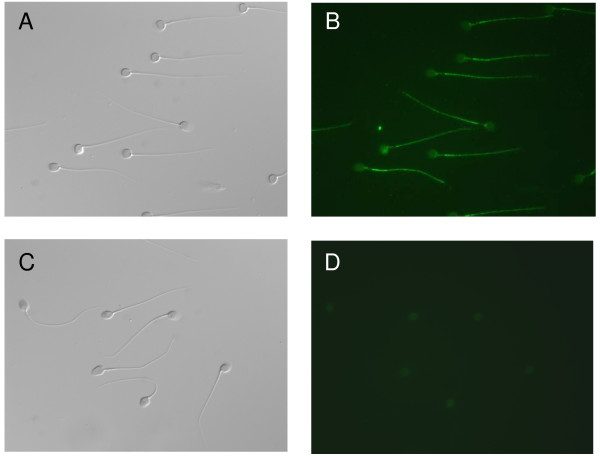
**Specificity of the Na_v_1.6 primary antibody**. Immunofluorescence (B, D) and corresponding phase-contrast image (A, C) of sperm cells stained with primary antibodies against Na_v_1.6 (1:500 dilution) in the absence (B) and presence (D) of immunogenic peptide (5 μg/ml).

Experiments were repeated at least three times with identical localization sites for each Na_v _channel in all cases. Immunogenic peptides were only available for Na_v_1.1, Na_v_1.3 and Na_v_1.6. Therefore, we could only assay the Na_v_1.6 one. Preincubation of the Na_v_1.6 primary antiserum (1:500 dilution) with the immunogenic peptide (5 μg/ml) caused an almost complete disappearance of the fluorescent signal (see Fig. 3). In addition, and for all Na_v _proteins assayed, unspecific binding was not observed in the two negative controls that were not exposed to the primary antibodies.

### Effects of the voltage-gated Na^+ ^channel agonist veratridine on human sperm motility

**Figure 4:**
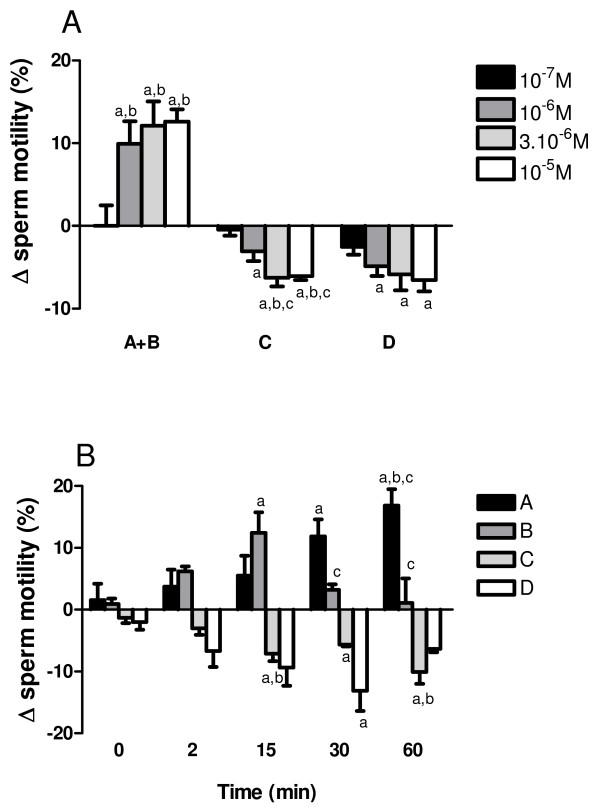
**Effects of veratridine on human sperm motility**. (A) Dose-response effects of veratridine (10^-7 ^M-10^-5 ^M) after 15 min incubation. Data represent percentage changes in sperm A + B, C and D motility grades between samples treated with veratridine and its solvent (paired controls). ^a^*P *< 0.05 versus responses in solvent-treated controls. ^b^*P *< 0.05 versus responses to veratridine 10^-7 ^M; ^*c*^*P *< 0.05 versus responses to veratridine 10^-6 ^M, one-way ANOVA. (B) Time-course of the effects of veratridine (3 × 10^-6 ^M) after 2, 15, 30 and 60 min incubation. Data represent percentage changes in sperm A, B, C and D motility grades between samples treated with veratridine and its solvent (paired controls). ^a^*P *< 0.05 versus responses at time 0; ^b^*P *< 0.05 versus responses at time 2, ^c^*P *< 0.05 versus responses at time 15, one-way ANOVA. Each bar is the mean with SEM of 6–12 different experiments.

Veratridine (10^-8 ^M-3 × 10^-5 ^M) caused time- and concentration-dependent increases in progressive motility (A + B grade) in capacitated sperm cells. The percentage of sperm A + B grades measured 5 min before (initial value) and 30 min after treatment with vehicle (paired controls) or veratridine, respectively, were: 62.7 ± 5.0, 61.5 ± 5.4 and 78.9 ± 3.1 for veratridine 10^-6 ^M (*n *= 12, *P *< 0.05, veratridine *vs *initial value and solvent-treated control), and 59.8 ± 5.6, 57.7 ± 7.2 and 82.3 ± 4.4 for veratridine 10^-5 ^M (*n *= 8, *P *< 0.05, veratridine *vs *initial value and solvent-treated control). Compared with vehicle-treated cells, the effects were significant at concentrations > 3 × 10^-7 ^M and were observed for at least 1 h of incubation (Fig. 4A, 4B).

The veratridine-induced rise in progressive motility increased slowly during the observation period (60 min) and was initially due mainly to an increase in the percentage of B grade cells (Fig. 4B). During the incubation time, this was replaced gradually by an increase in the percentage of A motility grade spermatozoa (Fig. 4B). The increase in progressive motility (A + B grade cells) was accompanied by a concomitant decrease in both C and D grade cells (Fig. 4).

### Effects of veratridine on intracellular free Ca^2+ ^concentration, [Ca^2+^]_i_

**Figure 5:**
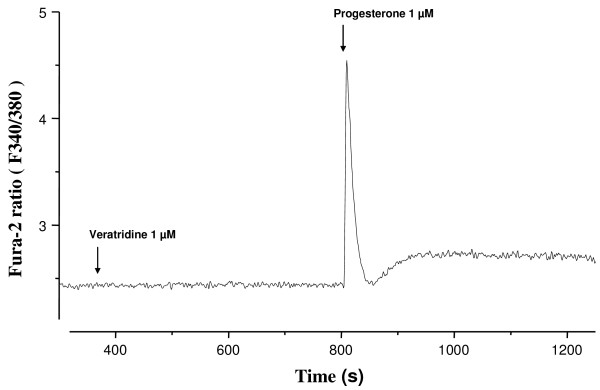
**Effects of veratridine (10^-6 ^M) and progesterone (10^-6 ^M) on intracellular free Ca^2+ ^levels, [Ca^2+^]_i_, in human sperm cells loaded with Fura-2**. The trace is representative of typical results obtained in 10 different experiments. The X Axis shows time in seconds with respect to addition of veratridine and progesterone and the Y axis shows [Ca^2+^]_i _data expressed by the ratio of F340/F380 signals.

Veratridine (10^-6 ^M) did not modify [Ca^2+^]_i _in Fura-2-loaded human sperm cells (Fig. 5). No effects were observed even after prolonged periods of incubation (30 min, not shown). Subsequent addition of progesterone (10^-6 ^M) to the same sperm aliquot caused a biphasic [Ca^2+^]_i _response consisting in a rapid transient peak followed by a decay to [Ca^2+^]_i _levels slightly over basal ones and a lower sustained plateau phase which persisted during the time of stimulation with progesterone (Fig. 5).

## Discussion

This study shows for the first time that voltage-dependent Na^+ ^channels are present, and at least some of them are functionally active, in human sperm cells. Ion channels play a central role in the regulation of sperm intra- and inter-cellular signaling [13-15,28-31]. The rapid ion fluxes through these membrane proteins permit a quick transfer of information between sperm and its surrounding [14,15]. This communication is essential for correct sperm guidance throughout the female reproductive tract as well as for acquisition of fertilization competence and interaction with the oocyte [12-17]. Many different ion channels have been identified in the sperm cell membrane. Among them, Ca^2+^, K^+ ^and anion channels are widely distributed in the head and flagellum and play an important role in regulating sperm function including motility, capacitation and acrosome reaction [13,14,17,28-31]. Na^+ ^channels should also be abundantly expressed in sperm, as the gradient of this ion across the plasma membrane plays a central role in the regulation of E_m_, a parameter that govern the rates and direction of ion-flow through channels and exchangers and modulates pH_i _[11,14,19]. It is well known that the process of capacitation is accompanied by important changes in sperm plasma membrane potential with a turn to a hyperpolarized state accompanied by an increase in pH_i _[13,14]. This hyperpolarization seems to be related with an increase in K^+ ^permeability and a decrease in Na^+ ^permeability [14,19,20]. In this context, the presence of epithelial Na^+ ^channels of the ENaC family has been demonstrated in sperm cells [19].

No studies have been made to identify the presence of voltage-dependent Na^+ ^channels in spermatozoa. This is probably due to the classical belief that these channels were present almost exclusively in nerves, skeletal muscle, and heart. As a consequence, little is known about the function of Na_v _channels in other tissues and cells, and particularly, at the reproductive level [9,27]. The present findings shows that the mRNAs encoding all known Na_v_α subunits and three β subunits are expressed in sperm cells. As sperm cells appear to be transcriptionally inactive, the mRNAs isolated from these cells would reflect gene expression processes that have taken place during earlier stages of spermatogenesis and, in good agreement, our results show that all VGSC mRNAs expressed in sperm were present in the human testis. The function of sperm mRNAs remains poorly understood [16,32,33]. A recent report has shown the existence of changes in the expression of several sperm proteins in the presence of a specific inhibitor of mitochondrial translation [34] indicating that, at least part of the mRNAs could be translated into proteins in sperm mitochondria [35] and play a role in the regulation of sperm physiology. Other sperm mRNAs could be transferred to the oocyte and be necessary for fertilization and/or for the initial steps of embryo development [32,33].

Immunofluorescence studies demonstrate that, with the exception of Na_v_1.1 and Na_v_1.3, all VGSC proteins could be detected in mature sperm. Moreover, the distribution of each Na_v _channel was very homogeneous within and throughout samples, with only minor changes in staining intensity. This wide expression and the specific and distinct distribution pattern of each Na_v _channel argue for an important role of VGSC in the regulation of sperm function. Some of them (i.e., Na_v_1.2, Na_v_1.4 and Na_v_1.7) were mainly found in the connecting piece, a region which play an important role in sperm signaling [17,36]. Of particular interest was the observation that Na_v_1.8 mRNA and protein are expressed in mature spermatozoa. Previous studies have suggested that this TTX-resistant channel is selectively expressed in a particular population of C-fiber and Aδ-fiber-associated sensory neurons and plays a key role in sensory transmission and pain perception [2,3,9]. The present data clearly shows that Na_v_1.8 is also expressed in cells of non-neuronal origin suggesting that, besides its role in nociception, Na_v_1.8 plays additional, still undefined roles in male reproduction. The localization of Na_v_1.8 in the flagellum and around the neck led us to hypothesize that it could be involved in modulation of flagellar activity and sperm motility.

In an attempt to investigate further the functional role of Na_v _channels in mature spermatozoa we analyzed the effect of the Na_v _activator veratridine on sperm motility. Veratridine caused time- and concentration-dependent increases in progressive motility. The effects of veratridine were characterized by an initial increase in B grade cells followed, after 15–30 min incubation, by a predominant increase in rapidly progressive A grade cells. This was accompanied by a decrease in sperm immotility with a reduction of C and D grade sperm cells. Veratridine acts by inhibiting Na_v _inactivation after spontaneous channel opening and, therefore, it is likely that effects could develop slowly, as channels spontaneously open and then become modified by veratridine. This mechanism of action could explain the gradual change in motility observed during the incubation time. Taken together, these data demonstrate that VGSCs participate in the regulation of human sperm motility.

The veratridine-induced increases in sperm progressive motility were not accompanied by any change in [Ca^2+^]_i_. In the same sperm samples, progesterone increased [Ca^2+^]_i _and caused the typical biphasic [Ca^2+^]_i _response, in accordance with previous reports [15,17,25,28]. The opening of Na_v _channels should produce a membrane depolarization with the subsequent opening of Ca^2+ ^channels or may cause an influx of Ca^2+ ^through the Na^+^/Ca^2+ ^exchanger acting in the reverse mode. Thus, the reasons why veratridine failed to modify [Ca^2+^]_i _remain unclear. A possible explanation is that veratridine-sensitive VGSCs may be mostly located in a particular tail segment [15,37] thus producing a low [Ca^2+^]_i _signal that could not be detected by fluorimetry or, alternatively, that opening of sperm VGSCs activate additional mechanisms that oppose Ca^2+ ^influx. In any case, our data strongly suggest that sperm motility could proceed in a Na^+^-dependent manner. The participation of a variety of Ca^2+^-dependent and Na^+^-dependent processes will ensure motility in a cell for which movement is essential to achieve its physiological function. This could explain the redundant expression of Na_v _and many other ion channels in sperm cells, as redundancy is not only a way to acquire new functions but also, and more important for a cell, for function conservation [38]. The use of Na^+ ^and Ca^2+ ^sources has additional advantages for the sperm cell since movement based on Na^+ ^currents should provide a more economic energy source than movement based exclusively on Ca^2+ ^currents [27]. Energy saving should be essential for a transcriptionally inactive small cell which must transverse the whole uterus and enter the oviduct to find its target cell, the oocyte. Finally, it is possible that the participation of Na^+^- and Ca^2+^-dependent mechanisms could vary during the sperm travel throughout the female reproductive tract depending on the degree of sperm activation and membrane potential state. In fact, sperm motility should be finely regulated to asseverate a good progressive motility while avoiding the development of hyperactivated motility that would led to a premature activation in an inappropriate place [12,17,22,36]. In this context, it is tempting to speculate that Na_v _channels could play a more important role in the non-capacitated and in the initial capacitation steps and be inactivated during capacitation, when sperm membrane hyperpolarizes previously to the acrosome reaction.

## Conclusion

This research shows the presence of voltage-dependent Na^+ ^channels in human sperm and supports a role for these channels in the regulation of mature sperm function. The data increase the diversity of ion channels expressed in spermatozoa and confirm the importance and complexity of sperm function regulation by ion channels.

## Abbreviations

VGSC: voltage-gated sodium channel; [Ca^2+^]_i_: intracellular free Ca^2+ ^levels; TTX: tetrodotoxin; pH_i_: intracellular pH; E_m_: plasma membrane potential; ENaC: epithelial sodium channel; BSA: bovine serum albumin; WHO: World Health Organization; mHTF: modified human tubal fluid; PBS: phosphate-buffered saline; ANOVA: analysis of variance.

## Competing interests

The authors declare that they have no competing interests.

## Authors' contributions

FMP carried out PCR and immunofluorescence experiments, participated in the design of the study and helped to write the manuscript. CGR and MFS participated in sample collection, capacitation and analysis of sperm parameters. MGC and ACR carried out motility studies and fluorimetric measurements. MLC wrote the manuscript and participated in the design of the study. All authors read and approved the final manuscript.
